# Ethnic variation in unexplained deaths in infancy, including sudden infant death syndrome (SIDS), England and Wales 2006–2012: national birth cohort study using routine data

**DOI:** 10.1136/jech-2018-210453

**Published:** 2018-07-04

**Authors:** Mary E Kroll, Maria A Quigley, Jennifer J Kurinczuk, Nirupa Dattani, Yangmei Li, Jennifer Hollowell

**Affiliations:** 1 Policy Research Unit in Maternal Health and Care, National Perinatal Epidemiology Unit, Nuffield Department of Population Health, University of Oxford, Oxford, UK; 2 School of Health Sciences, City, University of London, London, UK

**Keywords:** ethnicity, infant mortality, inequalities, cohort studies

## Abstract

**Background:**

Unexplained deaths in infancy comprise ‘sudden infant death syndrome’ (SIDS) and deaths without ascertained cause. They are typically sleep-related, perhaps triggered by unsafe sleep environments. Preterm birth may increase risk, and varies with ethnicity. We aimed to compare ethnic-specific rates of unexplained infant death, explore sociodemographic explanations for ethnic variation, and examine the role of preterm birth.

**Methods:**

We analysed routine data for 4.6 million live singleton births in England and Wales 2006–2012, including seven non-White ethnic groups ranging in size from 29 313 (Mixed Black-African-White) to 180 265 (Pakistani). We calculated rates, birth-year-adjusted ORs, and effects of further adjustments on the χ^2^ for ethnic variation.

**Results:**

There were 1559 unexplained infant deaths. Crude rates per 1000 live singleton births were as follows: 0.1–0.2 for Indian, Bangladeshi, Pakistani, White Non-British, Black African; 0.4 for White British; 0.6–0.7 for Mixed Black-African-White, Mixed Black-Caribbean-White, Black Caribbean. Birth-year-adjusted ORs relative to White British ranged from 0.38 (95% CI 0.24 to 0.60) for Indian babies to 1.73 (1.21 to 2.47) for Black Caribbean (χ^2^(10 df)=113.6, p<0.0005). Combined adjustment for parents’ marital/registration status and mother’s country of birth (UK/non-UK) attenuated the ethnic variation. Adjustments for gestational age at birth, maternal age and area deprivation made little difference.

**Conclusion:**

Substantial ethnic disparity in risk of unexplained infant death exists in England and Wales. Apparently not attributable to preterm birth or area deprivation, this may reflect cultural differences in infant care. Further research into infant-care practices in low-risk ethnic groups might enable more effective prevention of such deaths in the general population.

## Introduction

In 2015, about 7% of approximately 2500 annual deaths under the age of 1 year in England and Wales were ‘unexplained deaths in infancy’ (UDI),^1 2^ ie, recorded as ‘sudden infant death syndrome’ (SIDS, 60%) or without any ascertained cause (40%).^3 4^ Broadly equivalent to ‘sudden unexpected death in infancy’ (SUDI) and ‘sudden unexpected infant death’ (SUID),^5^ these deaths are typically ‘sleep-related’, occurring in circumstances consistent with failure of arousal in response to hypercarbia and hypoxia triggered by unsafe sleep environments.^6^ Risk factors identified in research studies include sleep position (front or side), inappropriate sleep surfaces, bed-sharing (particularly in combination with parental alcohol and/or drug use, or other risk factors), not sleeping in the same room as the parents, use of soft bedding, maternal smoking (prenatal or environmental) and not breastfeeding.^6 7^ Genetic alterations may increase vulnerability to UDI, but there is currently no evidence that causation has a strong heritable component.^7^

Striking ethnic disparities in rates of SUID or SUDI are reported in the USA^8^ and New Zealand.^9^ Until recently, there were no comparable National Statistics for England and Wales, because the data collected at birth and death registration did not include the ethnicity of either the baby or the mother. Since 2005, however, the Office for National Statistics has linked birth registrations with the ‘NHS Numbers for Babies’ dataset, which contains routine information recorded when the baby’s National Health Service number is generated, including the baby’s ethnicity (as reported by the mother) and gestational age at birth.^10 11^ Univariable analyses of births in 2005 found relatively high incidence of both preterm birth^11^ and SIDS^12^ among Black Caribbean babies in England and Wales, suggesting the possibility that ethnic variation in risk of UDI might be influenced by gestational age at birth. Preterm birth is associated with an increased risk of SIDS, and might be on the causal pathway, for example through incomplete development of the autonomic system that is responsible for arousal.^6^

We aimed to compare ethnic-specific national rates of UDI during 2006–2012, explore the extent to which sociodemographic factors might account for any ethnic disparities, and test the prior hypothesis that preterm birth might contribute to the relationship between ethnicity and risk of UDI.

## Methods

### Study design and data sources

We conducted a national birth cohort study, using routinely-collected administrative data. The study hypothesis arose before inspection of the data. The cohort consisted of all singletons born alive at 22+weeks’ gestation in England and Wales from 1 January 2006 to 31 December 2012. The Office for National Statistics provided an anonymised data extract linking birth and death registrations with the NHS Numbers for Babies dataset.^11^ Linkage methods and quality evaluation are described elsewhere.^10 13^ We further cleaned the data by removing records with missing or implausible data, as follows: gestation recorded as 43+weeks; birth weight missing; birth weight more than twice the IQR above or below the median within sex–gestation–ethnicity strata of the dataset; death not classifiable as explained or not; and maternal country of birth not classifiable as UK or non-UK.

### Variables

Following methodology used by the Office for National Statistics,^14^ based on the International Statistical Classification of Diseases and Related Health Problems, 10th revision (ICD-10), we defined UDI as death at age less than 1 year with either (a) any mention of R95 (SIDS) among the (up to 15) ICD-10 codes recording the causes of death, or (b) no mention of any cause except ICD-10 code R99 (’other ill-defined and unspecified causes of mortality').

Ethnicity coding in the NHS Numbers for Babies dataset is compatible with the categories used in the 2001 Census.^11^ We used the following ethnic groups: White British, White Non-British, Pakistani, Indian, Bangladeshi, Black African, Mixed Black-African-White, Black Caribbean, and Mixed Black-Caribbean-White. We created an ‘Other and Unspecific’ group by combining all the remaining stated categories, which were either very small (eg, Chinese) or only broadly defined (eg, ‘Any other Black background’). We included a separate ‘Unstated’ group.

Covariates were categorised as follows: gestational age at birth (22–31, 32–36, 37+weeks); baby’s sex (boy, girl); mother’s country of birth (UK, non-UK); mother’s age at delivery (<20, 20–24, 25–29, 30+years); parents’ marital/registration status based on the registration of the baby’s birth (sole registration, birth jointly registered by two parents living at different addresses, joint registration by two parents at the same address, birth within marriage)^11^; area deprivation based on the mother’s usual address at birth registration, measured as quintile categories of the respective national Index of Multiple Deprivation for England 2015^15^ and for Wales 2014.^16^

### Statistical analysis

We calculated crude rates of UDI per 1000 live singleton births by ethnic group over the full study period 2006–2012. We estimated risk for each ethnic group relative to White British by using a logistic regression model to calculate ORs with adjustment for potential confounding by birth year, which was fitted as a linear trend. To assess the extent to which other factors in our dataset might account for the ethnic variation in risk, we fitted exploratory models that were adjusted for these covariates first individually and then cumulatively, choosing each additional factor to minimise the χ^2^ for ethnic variation, based on the likelihood ratio test statistic. To avoid potential problems with sparse data, we did not include interaction terms. All analyses were done in Stata V.13, using two-sided tests with a 5% significance level.

### Sensitivity analyses

We treated quintile categories of area deprivation for England and for Wales as equivalent in the main analysis; to check sensitivity to this simplification, the analysis was repeated using a 10-level factor representing each combination of England/Wales and the five quintiles. We did not include birth weight in the main analysis because it is very closely associated with gestation; for completeness, the analysis was repeated replacing gestation with birth weight, expressed either as categories (<1.5 kg, 1.5–<2.5 kg, 2.5–<4 kg, 4+kg) or as ‘small for gestational age’ (10% cut-off within sex–gestation strata of the dataset). To check for sparse-data bias, which can occur in maximum likelihood estimation where there are small numbers of events in some categories,^17^ the analysis was repeated using a penalised-likelihood method.^18^

### Public involvement

Representatives of organisations concerned with infant health and/or work related to ethnic minority groups were consulted at the design and interpretation phases of the study and will be involved in dissemination of the results.

## Results

The source file contained data on 4 744 666 babies. We successively excluded 16 695 for implausible gestation, 20 999 for missing birth weight, and 72 040 for implausible birth weight, leaving 4 634 932. Of the 15 001 infant deaths in the reduced dataset, 75 deaths had no ICD-10 cause codes. For these deaths, we used the ‘underlying cause’ if supplied (38 postneonatal deaths, nearly all in babies born in 2007, and 12 neonatal deaths, all in babies born in 2012).^19^ We were unable to classify the remaining 25 neonatal deaths as explained or unexplained and excluded these babies (all born in 2007) from the analysis. We further excluded 143 babies whose mother’s country of birth could not be classified as UK or non-UK. After exclusions (2.3%), 4 634 764 babies remained for analysis (table 1); the proportion of births with unstated ethnicity was 6%, decreasing from 10% in 2006 to 3% in 2012, and there were no other missing data.

**Table 1:** **Risk of unexplained death in infancy, by ethnic group and other factors**. Live singleton births at 22+weeks, England and Wales 2006–2012

Factor	Births		Unexplained infant deaths		Rate (95% CI)	OR (95% CI)
	N	%	n	%	Crude per 1000	Adjusted for birth year
**Ethnic group**						
White British (ref)	3 009 144	65	1129	72	0.38 (0.35 to 0.40)	1
White Non-British	340 515	7	63	4	0.19 (0.14 to 0.24)	**0.50 (0.39 to 0.64)**
Pakistani	180 265	4	33	2	0.18 (0.13 to 0.26)	**0.49 (0.35 to 0.69)**
Indian	132 646	3	19	1	0.14 (0.09 to 0.22)	**0.38 (0.24 to 0.60)**
Bangladeshi	62 944	1	10	1	0.16 (0.08 to 0.29)	**0.42 (0.23 to 0.79)**
Black African	154 071	3	37	2	0.24 (0.17 to 0.33)	**0.64 (0.46 to 0.89)**
Mixed Black-African-White	29 313	1	17	1	0.58 (0.34 to 0.93)	1.55 (0.96 to 2.50)
Black Caribbean	47 503	1	31	2	0.65 (0.44 to 0.93)	**1.73 (1.21 to 2.47)**
Mixed Black-Caribbean-White	46 445	1	28	2	0.60 (0.40 to 0.87)	**1.62 (1.11 to 2.36)**
Other/unspecific	344 186	7	92	6	0.27 (0.22 to 0.33)	**0.72 (0.58 to 0.88)**
Unstated	287 732	6	100	6	0.35 (0.28 to 0.42)	0.89 (0.73 to 1.10)
**Parent marital/registration status**						
Within marriage (ref)	2 499 006	54	388	25	0.16 (0.14 to 0.17)	1
Joint registration, same address	1 398 905	30	570	37	0.41 (0.37 to 0.44)	**2.64 (2.32 to 3.01)**
Joint registration, different addresses	450 482	10	309	20	0.69 (0.61 to 0.77)	**4.46 (3.84 to 5.18)**
Sole registration	286 371	6	292	19	1.02 (0.91 to 1.14)	**6.55 (5.63 to 7.62)**
**Mother country of birth**						
Non-UK (ref)	1 127 462	24	190	12	0.17 (0.15 to 0.19)	1
UK	3 507 302	76	1369	88	0.39 (0.37 to 0.41)	**2.30 (1.98 to 2.68)**
**Deprivation, IMD 5ths**						
1=Advantaged (ref)	708 738	15	109	7	0.15 (0.13 to 0.19)	1
2	771 227	17	152	10	0.20 (0.17 to 0.23)	**1.28 (1.00 to 1.64)**
3	862 148	19	231	15	0.27 (0.23 to 0.30)	**1.75 (1.39 to 2.20)**
4	1 031 689	22	399	26	0.39 (0.35 to 0.43)	**2.53 (2.04 to 3.12)**
5=Disadvantaged	1 260 962	27	668	43	0.53 (0.49 to 0.57)	**3.46 (2.82 to 4.23)**
**Gestation, weeks**						
37+ (ref)	4 376 271	94	1269	81	0.29 (0.27 to 0.31)	1
32–36	217 808	5	224	14	1.03 (0.90 to 1.17)	**3.54 (3.07 to 4.08)**
22–31	40 685	1	66	4	1.62 (1.25 to 2.06)	**5.57 (4.35 to 7.13)**
**Mother age, years**						
30+ (ref)	2 206 398	48	429	28	0.19 (0.18 to 0.21)	1
25–29	1 265 682	27	393	25	0.31 (0.28 to 0.34)	**1.60 (1.39 to 1.83)**
20–24	886 825	19	465	30	0.52 (0.48 to 0.57)	**2.69 (2.36 to 3.07)**
<20	275 859	6	272	17	0.99 (0.87 to 1.11)	**5.03 (4.32 to 5.85)**
**Sex**						
Girl (ref)	2 257 086	49	617	40	0.27 (0.25 to 0.30)	1
Boy	2 377 678	51	942	60	0.40 (0.37 to 0.42)	**1.45 (1.31 to 1.60)**
**Total**	4 634 764	100	1559	100	0.34 (0.32 to 0.35)	–

Number of live births (N, %); number of unexplained infant deaths (n, %); crude rate per 1000 live singleton births with 95% CI by ethnic group and other factors; OR relative to reference category (adjusted only for birth year). ORs shown in bold have CIs that do not include 1. Likelihood ratio test for ethnic variation (adjusted only for birth year): χ^2^(10 df)=113.6, p<0.0005.

IMD, Index of Multiple Deprivation.

**Figure 1:**
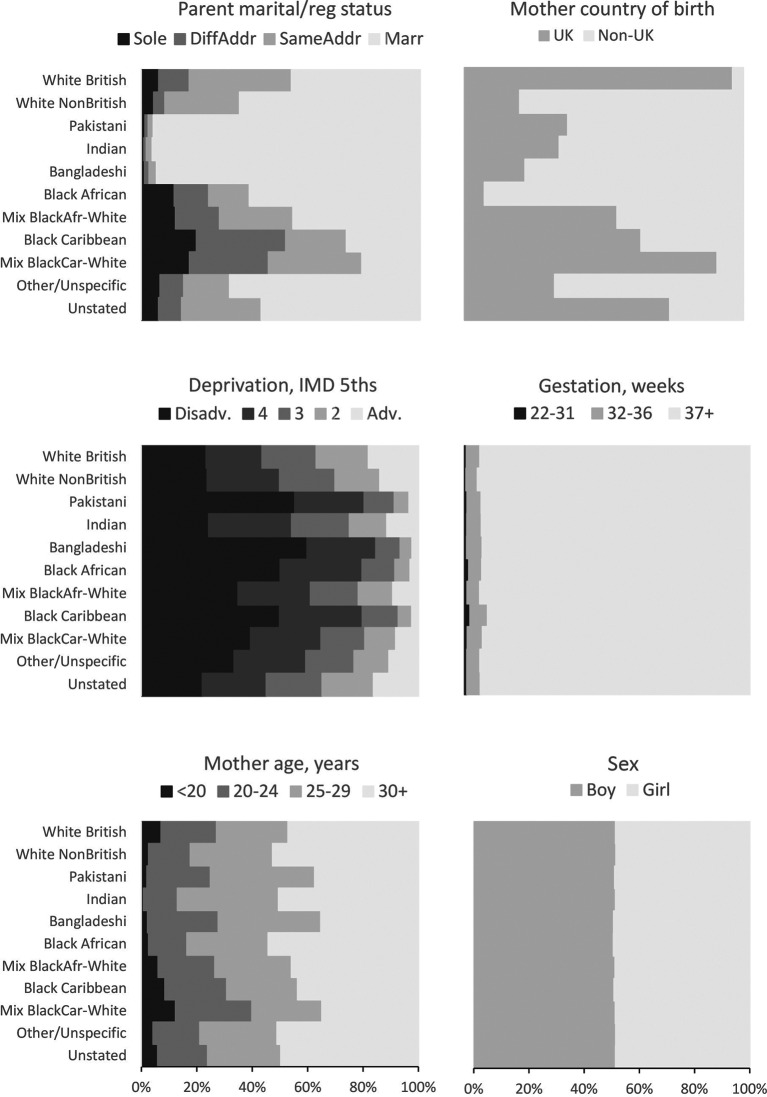
**Distributions of covariates by ethnic group.** Live singleton births at 22+weeks, England and Wales 2006–2012. Data are shown in online supplementary table S1. IMD, Index of Multiple Deprivation.

Figure 1 shows the distributions of maternal and infant sociodemographic characteristics by ethnic group (data in online supplementary table S1). The proportion of births registered outside marriage was low (4%–5%) for all three South Asian groups, and high for the Black Caribbean (74%) and Mixed Black-Caribbean-White (79%) groups. The proportion of mothers born in the UK was low for the Black African group (7%), and high for Mixed Black-Caribbean-White (91%) and White British (96%). The proportion of births in the most deprived quintile was 23%–24% for the White British, White Non-British and Indian groups, and 50%–60% for the Black Caribbean, Black African, Pakistani and Bangladeshi groups. The proportion of preterm births was higher for Black Caribbean babies (8%) than others (5%–6%).

10.1136/jech-2018-210453.supp1 Supplementary file 1

**Figure 2:**
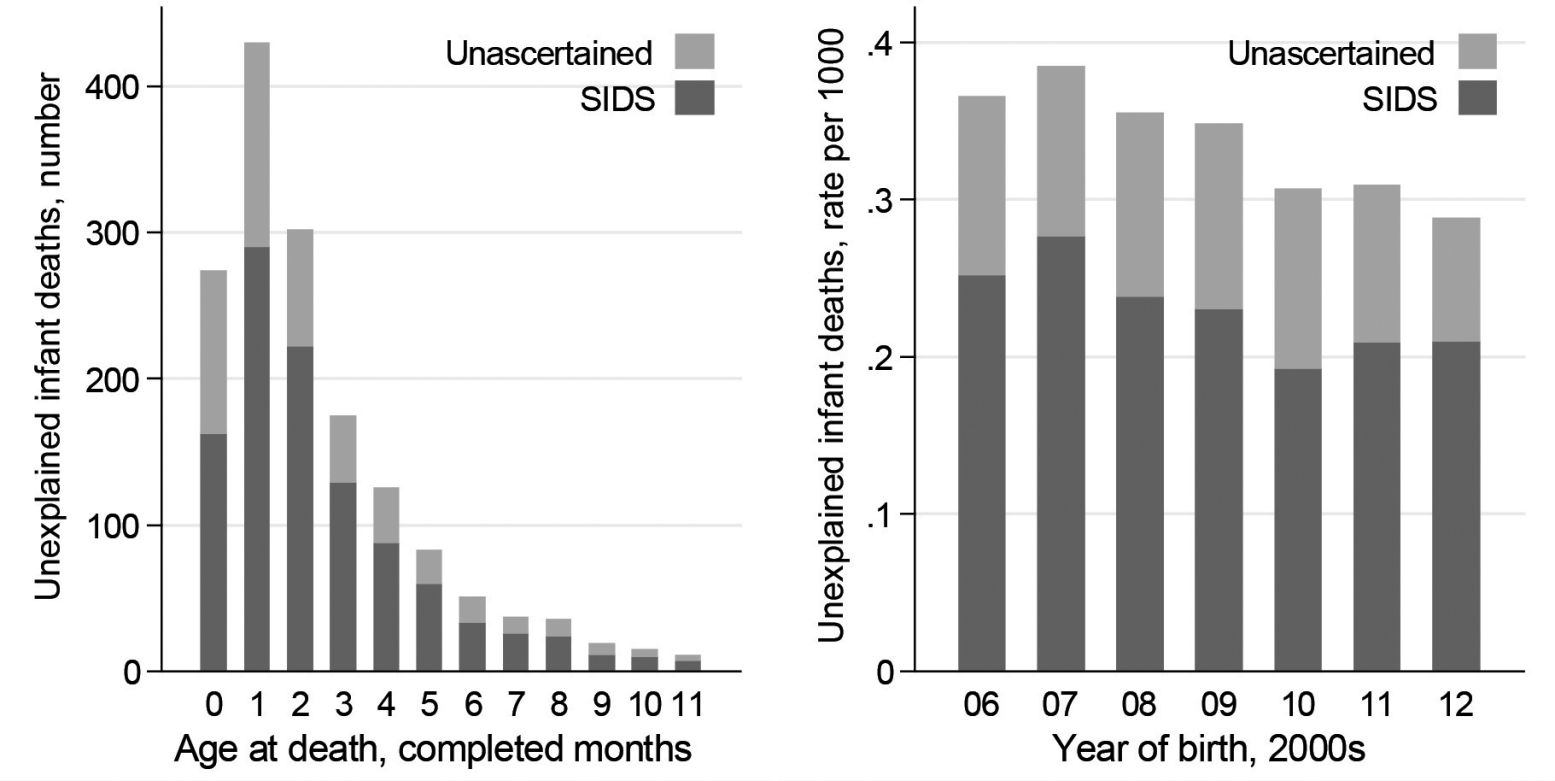
**Unexplained deaths in infancy, by recorded cause.** Number of unexplained deaths in infancy, by age at death. Crude rate of unexplained infant death per 1000, by year of birth. Live singleton births at 22+weeks, England and Wales 2006–2012. SIDS, sudden infant death syndrome. Data are shown in online supplementary table S2.

There were 1559 UDIs, with highest frequency in the second month after birth (figure 2; data in online supplementary table S2). The proportion coded as SIDS (68% overall) varied little with age at death or with birth year. Each covariate was associated with the risk of UDI in the general population after adjustment for potential confounding by birth year (table 1): risk was greater for babies born outside marriage (with risk increasing over joint registration by two parents living at the same address, joint registration at different addresses and sole registration), UK-born mothers, higher levels of area deprivation, male babies, younger mothers and earlier gestational age at birth.

**Figure 3:**
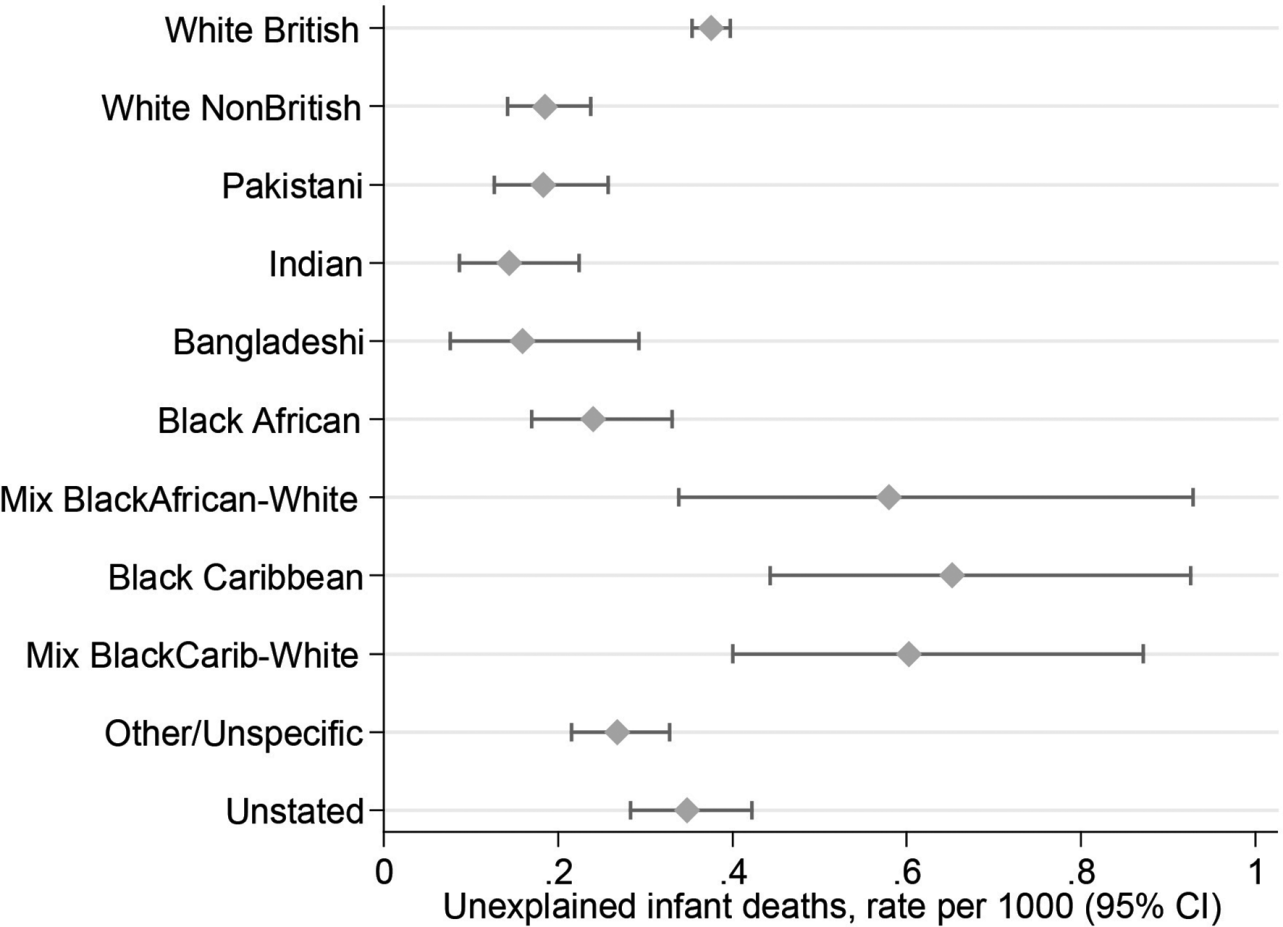
**Unexplained death in infancy, by ethnic group.** Crude rate of unexplained infant death (95% CI) per 1000 live singleton births at 22+weeks, England and Wales 2006–2012. Data are shown in table 1.

The crude rate of UDI was 0.34 per 1000 live singleton births, varying from 0.1–0.2 for the South Asian, White Non-British and Black African groups, to 0.4 for White British, and to 0.6–0.7 for Black Caribbean and both Mixed Black-White groups (table 1, figure 3). The highest rate (Black Caribbean) was 4.6 times the lowest (Indian). Adjusting for birth year, there was statistically significant ethnic variation in risk (χ^2^(10 df)=113.6, p<0.0005), with ORs relative to White British ranging from 0.38 (95% CI 0.24 to 0.60) for Indian babies to 1.73 (95% CI 1.21 to 2.47) for Black Caribbean (table 1).

In exploratory analyses, the ethnic variation in risk of UDI remained statistically significant when the ORs were adjusted for each covariate individually; the residual ethnic variation was least after adjustment for parents’ marital/registration status (χ^2^=26.7, p=0.003) and greatest after adjustment for deprivation (χ^2^=163.3, p<0.0005) (table 2). When the factors were fitted cumulatively, in the order that minimised the χ^2^ for ethnic variation at each stage, the ethnic variation ceased to be statistically significant after joint adjustment for parental marital/registration status and maternal country of birth (χ^2^=7.8, p=0.6); with these two adjustments none of the ethnic groups was significantly different from White British (table 2). Further adjustments for deprivation, sex, gestation and maternal age made little difference.

**Table 2:** **Exploratory analyses.** Covariate-adjusted ORs for unexplained death in infancy, by ethnic group relative to White British. Live singleton births at 22+weeks, England and Wales 2006–2012

Ethnic group	OR (95% CI)	OR (95% CI)	OR (95% CI)	OR (95% CI)	OR (95% CI)	OR (95% CI)
With individual adjustment for these factors:						
**Individual models**	**Parent marital/** **registration status**	**Mother COB**	**Mother age**	**Gestation**	**Sex**	**Deprivation**
White British	1	1	1	1	1	1
White Non-British	**0.63 (0.49 to 0.81)**	0.88 (0.66 to 1.16)	**0.57 (0.44 to 0.73)**	**0.51 (0.40 to 0.66)**	**0.50 (0.39 to 0.64)**	**0.48 (0.37 to 0.61)**
Pakistani	0.97 (0.68 to 1.38)	0.74 (0.52 to 1.05)	**0.51 (0.36 to 0.71)**	**0.48 (0.34 to 0.68)**	**0.49 (0.35 to 0.69)**	**0.35 (0.25 to 0.49)**
Indian	0.78 (0.49 to 1.23)	**0.60 (0.38 to 0.94)**	**0.46 (0.29 to 0.73)**	**0.38 (0.24 to 0.60)**	**0.38 (0.24 to 0.60)**	**0.35 (0.22 to 0.56)**
Bangladeshi	0.83 (0.44 to 1.55)	0.74 (0.39 to 1.39)	**0.43 (0.23 to 0.80)**	**0.42 (0.22 to 0.78)**	**0.42 (0.23 to 0.79)**	**0.29 (0.16 to 0.55)**
Black African	**0.64 (0.46 to 0.89)**	1.30 (0.90 to 1.88)	0.74 (0.54 to 1.03)	**0.62 (0.45 to 0.87)**	**0.64 (0.46 to 0.89)**	**0.47 (0.34 to 0.65)**
Mixed Black-African-White	1.36 (0.84 to 2.19)	**2.04 (1.26 to 3.30)**	1.57 (0.97 to 2.53)	1.55 (0.96 to 2.50)	1.55 (0.96 to 2.51)	1.31 (0.81 to 2.12)
Black Caribbean	1.14 (0.79 to 1.63)	**2.13 (1.49 to 3.05)**	**1.65 (1.15 to 2.36)**	**1.60 (1.12 to 2.29)**	**1.73 (1.21 to 2.48)**	1.26 (0.88 to 1.80)
Mix Black-Caribbean-White	1.09 (0.75 to 1.58)	**1.67 (1.15 to 2.43)**	1.37 (0.94 to 2.00)	**1.58 (1.09 to 2.30)**	**1.62 (1.11 to 2.36)**	1.32 (0.91 to 1.93)
Other/unspecific	0.85 (0.69 to 1.05)	1.12 (0.89 to 1.42)	0.78 (0.63 to 0.96)	0.71 (0.58 to 0.88)	0.71 (0.58 to 0.88)	0.62 (0.50 to 0.76)
Unstated	0.98 (0.80 to 1.21)	1.03 (0.83 to 1.26)	0.94 (0.76 to 1.15)	0.89 (0.73 to 1.10)	0.89 (0.73 to 1.10)	0.89 (0.72 to 1.09)
χ^2^ for ethnic variation	26.7	42.2	78.7	111.3	113.5	163.3
P value	**0.003**	**<0.0005**	**<0.0005**	**<0.0005**	**<0.0005**	**<0.0005**
With cumulative adjustments in the following order to minimise the χ^2^ for ethnic variation at each stage:						
**Cumulative models**	**Parent marital/** **registration status**	**+Mother COB**	**+Deprivation**	**+Sex**	**+Gestation**	**+Mother age**
White British	1	1	1	1	1	1
White Non-British	**0.63 (0.49 to 0.81)**	0.91 (0.69 to 1.21)	0.90 (0.68 to 1.20)	0.90 (0.68 to 1.20)	0.90 (0.68 to 1.20)	0.92 (0.69 to 1.23)
Pakistani	0.97 (0.68 to 1.38)	1.24 (0.86 to 1.78)	0.94 (0.65 to 1.36)	0.94 (0.65 to 1.36)	0.92 (0.64 to 1.33)	0.87 (0.60 to 1.26)
Indian	0.78 (0.49 to 1.23)	1.02 (0.64 to 1.63)	0.91 (0.57 to 1.46)	0.91 (0.57 to 1.46)	0.88 (0.55 to 1.42)	0.87 (0.54 to 1.39)
Bangladeshi	0.83 (0.44 to 1.55)	1.15 (0.61 to 2.17)	0.87 (0.46 to 1.64)	0.87 (0.46 to 1.65)	0.85 (0.45 to 1.61)	0.79 (0.42 to 1.50)
Black African	**0.64 (0.46 to 0.89)**	1.02 (0.70 to 1.48)	0.90 (0.62 to 1.30)	0.90 (0.62 to 1.31)	0.88 (0.61 to 1.28)	0.96 (0.66 to 1.40)
Mixed Black-African-White	1.36 (0.84 to 2.19)	1.59 (0.98 to 2.57)	1.52 (0.94 to 2.47)	1.52 (0.94 to 2.47)	1.54 (0.95 to 2.50)	1.59 (0.98 to 2.57)
Black Caribbean	1.14 (0.79 to 1.63)	1.31 (0.91 to 1.88)	1.17 (0.82 to 1.69)	1.18 (0.82 to 1.69)	1.13 (0.79 to 1.63)	1.22 (0.85 to 1.75)
Mix Black-Caribbean-White	1.09 (0.75 to 1.58)	1.11 (0.76 to 1.62)	1.05 (0.72 to 1.53)	1.05 (0.72 to 1.53)	1.05 (0.72 to 1.54)	1.06 (0.73 to 1.55)
Other/unspecific	0.85 (0.69 to 1.05)	1.09 (0.87 to 1.37)	1.02 (0.81 to 1.28)	1.02 (0.81 to 1.28)	1.00 (0.80 to 1.26)	1.02 (0.81 to 1.28)
Unstated	0.98 (0.80 to 1.21)	1.06 (0.86 to 1.30)	1.07 (0.87 to 1.31)	1.07 (0.87 to 1.31)	1.06 (0.86 to 1.30)	1.06 (0.86 to 1.31)
χ^2^ for ethnic variation	26.7	7.8	5.1	5.1	5.1	6.4
P value	**0.003**	0.6	0.9	0.9	0.9	0.8

All models are also adjusted for birth year. ‘COB’, country of birth (non-UK/UK). ‘χ^2^ for ethnic variation’, likelihood ratio test χ^2^ (10 df). ORs shown in bold have CIs that do not include 1.

Sensitivity analyses did not materially affect the findings. Adjustments for birth weight or ‘small for gestational age’ were less effective than adjustment for gestation in reducing the χ^2^ for ethnic variation. Modelling the deprivation scores for England and Wales separately, or using penalised rather than maximum likelihood methods, made little difference.

## Discussion

### Statement of principal findings

There is nearly fivefold variation in risk of UDI between ethnic groups in England and Wales. This variation does not appear to be explained by preterm birth, baby’s sex, maternal age, or area deprivation. The risk is lowest for Indian, Bangladeshi, Pakistani, White Non-British and Black African babies; intermediate for White British; and highest for Mixed Black-African-White, Mixed Black-Caribbean-White and Black Caribbean. Parents’ marital/registration status (married, joint registration by two parents living at the same address, joint registration with different addresses and sole registration by one parent) and mother’s country of birth (non-UK/UK) seem to jointly account for much of the ethnic disparity in risk, perhaps representing cultural variation in causal factors.

### Strengths and weaknesses of the study

This is the first study to quantify risk of UDI in England and Wales by ethnicity. Based on a recent 7-year national birth cohort, the study is large enough to yield precise estimates for relatively homogeneous minority ethnic groups. Some of the findings may be generalisable to similar groups resident in other countries. Several potentially important covariates are examined. Studies based on routine data are particularly valuable for this research area, because much of the existing evidence for risk factors derives from interview-based case–control studies, with potential for participation and reporting bias. The study is limited by a lack of information for individual families on infant sleep practices and other lifestyle factors, although prior national surveys provide some relevant data at the subpopulation level, as discussed below. The proportion of births with unstated ethnicity is 6% overall, decreasing with time, but the characteristics of such births have been shown to resemble those of White British,^11^ and our analysis is adjusted for potential confounding by birth year. We are unable to generalise from the White Non-British group because it is very diverse: maternal countries of birth include Poland (24%); UK (20%); Lithuania and Romania (4% each); South Africa and Turkey (3% each); France, Slovakia, Ireland, the USA, Germany, Latvia, Portugal, Australia, Czech Republic (2% each); and >200 other countries worldwide. Social disadvantage is measured by area-based deprivation, which does not necessarily reflect individual socioeconomic status. Although the ethnic group recorded in the NHS Numbers for Babies dataset is nominally the ethnicity of the baby as reported by the mother to the health professional notifying the birth, in practice it may sometimes have been the ethnicity of the mother or decided by the health professional without asking the mother.^11^ Any resulting misclassification might tend to underestimate differences between ethnic groups; the size of this potential bias is uncertain but probably small.

### Relation to other studies

To our knowledge, there are only three comparable UK studies.^12 20 21^ All examined SIDS (not UDI), and two date from the 1980s: one of these was regional (not national) and the other used mother’s country of birth as a surrogate for ethnicity. A univariable analysis of ethnic variation in infant mortality for babies born in England and Wales in 2005 noted higher risk of SIDS in Caribbean babies.^12^ In Birmingham (England) 1981-83, ethnic differences in risk of SIDS (low for Asian and high for Afro-Caribbean babies) persisted after controlling for social class, birth weight and maternal age,^21^ consistent with our finding that individual adjustment for area deprivation, gestation and maternal age made little difference to the ethnic variation. In England and Wales 1982-85, babies of mothers born in Bangladesh, West Africa, East Africa, India, Pakistan and the Caribbean had lower SIDS rates than babies of UK-born mothers^20^; in our study, Black Caribbean babies had higher UDI rates than White British, but their mothers were mostly born in the UK. Internationally, our findings are consistent with a pattern of relatively low risk for Asian groups (i.e. originating in East, South East or South Asia) within majority White populations, as reported in USA 2011-14,^8^ New Zealand 2003-07^9^ and (using maternal country of birth) Victoria (Australia) 1985-89.^22^ Bearing in mind potential differences in culture, our results for the Black Caribbean and Mixed Black-White groups also appear consistent with the relatively high risk of SUID reported for Black Non-Hispanic babies in the USA.^8^

### Meaning of the study

In the UK from the mid-1950s to the late 1980s, professional advice to use the front or side sleeping position influenced a whole generation of mothers, and was almost certainly a real cause of SIDS.^23^ Subsequent public health campaigns have aimed to correct this advice, stressing sleep position among other aspects of ‘safe sleep’.^24^ Nevertheless, a recent multiagency review set in the West Midlands (England) reported that ‘most SUDI still occur in hazardous sleep environments, despite public health campaigns’.^25^ This suggests that, among ethnic groups with high proportions of UK-born mothers, prevalence of unsafe sleep practices may be associated with inaccessibility to health messages, and hence perhaps with sociodemographic characteristics. Parental marital/registration status, in particular, may represent a constellation of risk factors: a survey of maternity-care users in England in 2007 found that single mothers were less likely to access timely care, attend NHS antenatal classes, or have a postnatal check-up.^26^ Unfortunately, direct evidence on relevant infant care practices is very limited: for example, a comparison of 20 Afro-Caribbean with 33 White British babies in East London found that the Afro-Caribbean babies were more likely to share the parental bed and to ‘prefer’ front sleeping, but numbers were very small, the participating parents were volunteers and more than one preferred sleep position could be reported, with no indication of frequency.^27^

Protective infant-care traditions may tend to persist in ethnic groups with high proportions of mothers born outside the UK. In 1991, shortly before the start of the national ‘Back to Sleep’ campaign, a survey of multiparous mothers attending an antenatal clinic in Birmingham found that 34% of 172 South Asian mothers, or 47% of those who had lived in the UK for less than 5 years, reported placing their babies on their backs for sleep, compared with 6% of 202 White mothers.^28^ The authors commented that the South Asians were probably continuing practices learnt from previous generations, facilitated by the active involvement of older members of the extended family. Similarly, a Welsh qualitative study found that White babies might sleep in their parent’s room for a few months but would then be encouraged to ‘get used’ to sleeping alone, whereas Bangladeshi babies were less likely to be alone at any time and would remain with adults during daytime sleeping.^29^ In Bradford (England) 2008-09, a large telephone survey found that Pakistani parents were more likely than White British to report that their babies bed-shared or used a pillow; however, reported alcohol use was rare,^30^ and a qualitative study in the same population found that the typical ‘pillow’ was a traditional small firm headrest designed to prevent flattening of the skull while sleeping on the back, not a soft European-style pillow.^31^ It has been suggested that similar small headrests used in East Asia may reduce the risk of rolling over to the front position.^32^ Further research is needed, bearing in mind current concern about the safety of ‘infant sleep positioners’.^7 33 34^

Although maternal smoking and not breast feeding are associated with increased risk of sleep-related infant death in the general population,^7^ the ethnic patterns of tobacco use and breast feeding seen in UK national survey data do not seem entirely consistent with the ethnic pattern of UDI seen in our study. Black Caribbean mothers in the Millennium Cohort Study (MCS) had a higher rate of breast feeding initiation,^35^ and a lower rate of discontinuation before 4 months,^36^ than any other ethnic group except Black African and ‘Other White’, whereas Black Caribbean babies in our study had the highest risk of UDI. Self-reported current smoking in the MCS was rare among South Asian (particularly Bangladeshi) and Black African mothers, and relatively frequent among ‘Other White’, White British and Black Caribbean mothers.^37^ In the 2004 Health Survey for England, however, young Bangladeshi women reported chewing (not smoking) tobacco, and had higher prevalence of cotinine in saliva assays than young Pakistani, Indian or African women, while Bangladeshi men had higher prevalence of cigarette smoking, and cotinine, than men in any other minority ethnic group or the general population.^38^ Thus, Bangladeshi babies could be exposed to components of tobacco both prenatally (through their mothers’ chewing and secondhand smoking) and postnatally (through environmental smoke). Against this background, Bangladeshi babies in our study had the second-lowest risk of UDI.

### Policy implications and future research

Safe-sleep campaigns in the UK should target the ethnic groups that experience higher rates of UDI. Further research into ethnic-specific infant care practices is required, and potentially protective customs in South Asian families should be evaluated. Implications for the aetiology of UDI should be considered.

**What is already known on this subject**

- Striking ethnic disparities in risks of ‘sudden unexpected infant death’ (SUID) or ‘sudden unexpected death in infancy’ (SUDI) exist in the USA and New Zealand.
- Comparable UK data have recently become available, making it possible for the first time to investigate national variation in risk among the ethnic groups resident in England and Wales.

**What this study adds**

- There is nearly fivefold variation between ethnic groups in England and Wales in risk of ‘sudden infant death syndrome’ (SIDS) and other unexplained death in infancy.
- The variation does not seem to be attributable to preterm birth, maternal age or area deprivation, and may reflect cultural differences in infant care.
- Further research into infant-care practices in low-risk ethnic groups might enable more effective prevention of these deaths in the general population.

## References

[1] Office for National Statistics. Childhood mortality in England and Wales: 2015. Statistical Bulletin. 2017 https://www.ons.gov.uk/peoplepopulationandcommunity/birthsdeathsandmarriages/deaths/bulletins/childhoodinfantandperinatalmortalityinenglandandwales/2015 (accessed Nov 2017).

[2] Office for National Statistics. Unexplained Deaths in Infancy, England and Wales: 2015. Statistical Bulletin. 2017 https://www.ons.gov.uk/peoplepopulationandcommunity/birthsdeathsandmarriages/deaths/bulletins/unexplaineddeathsininfancyenglandandwales/2015 (accessed Nov 2017).

[3] Willinger M, James LS, Catz C Defining the sudden infant death syndrome (SIDS): deliberations of an expert panel convened by the National Institute of Child Health and Human Development. Pediatr Pathol 1991;11:677–84. 10.3109/15513819109065465 1745639

[4] Corbin T Investigation into sudden infant deaths and unascertained infant deaths in England and Wales, 1995-2003. Health Stat Q 2005:17–23. 16138751

[5] Taylor BJ, Garstang J, Engelberts A, et al International comparison of sudden unexpected death in infancy rates using a newly proposed set of cause-of-death codes. Arch Dis Child 2015;100:1018–23. 10.1136/archdischild-2015-308239 26163119

[6] Carlin RF, Moon RY Risk factors, protective factors, and current recommendations to reduce sudden infant death syndrome: a review. JAMA Pediatr 2017;171:175–80. 10.1001/jamapediatrics.2016.3345 27918760

[7] Moon RY AAP Task Force on Sudden Infant Death Syndrome. SIDS and other sleep-related infant deaths: Evidence base for 2016 updated recommendations for a safe infant sleeping environment. Pediatrics 2016;138:e20162940. 27940805 10.1542/peds.2016-2940

[8] Centers for Disease Control and Prevention. Sudden unexpected infant death and sudden infant death syndrome. Data and statistics. 2017 https://www.cdc.gov/sids/data.htm (accessed Apr 2017).

[9] Child and Youth Mortality Review Committee. Fifth report to the minister of health: Reporting mortality 2002–2008. 2009 http://www.cymrc.health.govt.nz (accessed Apr 2017).

[10] Hilder L, Moser K, Dattani N, et al Pilot linkage of NHS numbers for babies data with birth registrations. Health Stat Q 2007;33:25–33. 17373380

[11] Moser K, Stanfield KM, Leon DA Birthweight and gestational age by ethnic group, England and Wales 2005: introducing new data on births. Health Stat Q 2008;39:22–31. 18810886

[12] Gray R, Headley J, Oakley L, et al Towards an understanding of variations in infant mortality rates between different ethnic groups. Inequalities in Infant Mortality Project: Briefing Paper 3. Oxford: National Perinatal Epidemiology Unit, 2009.

[13] Office for National Statistics. Revisions to gestation-specific Infant Mortality data set 2007-2012. Information note. 2015 www.ons.gov.uk/ons/guide-method/user-guidance/health-and-life-events/revisions-to-gestation-specific-infant-mortality-data-set-2007-to-2012.pdf (accessed Apr 2017).

[14] Office for National Statistics. Unexplained deaths in infancy: England and Wales QMI. Methodology. 2017 https://www.ons.gov.uk/peoplepopulationandcommunity/birthsdeathsandmarriages/deaths/methodologies/unexplaineddeathsininfancyenglandandwalesqmi (accessed Nov 2017).

[15] Smith T, Noble M, Noble S, et al The English Indices of Deprivation 2015. Technical Report. 2015 https://www.gov.uk/government/uploads/system/uploads/attachment_data/file/464485/English_Indices_of_Deprivation_2015_-_Technical-Report.pdf (accessed Sep 2017).

[16] Welsh Government. Welsh Index of Multiple Deprivation (WMD) 2014 Revised. 2014 http://gov.wales/statistics-and-research/welsh-index-multiple-deprivation/?lang=en (accessed Sep 2017).

[17] Greenland S, Mansournia MA, Altman DG Sparse data bias: a problem hiding in plain sight. BMJ 2016;352:i1981 10.1136/bmj.i1981 27121591

[18] Firth D Bias reduction of maximum likelihood estimates. Biometrika 1993;80:27–38. 10.1093/biomet/80.1.27

[19] Dattani N, Rowan S Causes of neonatal deaths and stillbirths: a new hierarchical classification in ICD-10. Health Stat Q 2002;15:16–22.

[20] Balarajan R, Soni Raleigh V, Botting B Sudden infant death syndrome and postneonatal mortality in immigrants in England and Wales. BMJ 1989;298:716–20. 10.1136/bmj.298.6675.716 2496819 PMC1836015

[21] Kyle D, Sunderland R, Stonehouse M, et al Ethnic differences in incidence of sudden infant death syndrome in Birmingham. Arch Dis Child 1990;65:830–3. 10.1136/adc.65.8.830 2400217 PMC1792482

[22] Kilkenny M, Lumley J Ethnic differences in the incidence of the sudden infant death syndrome (SIDS) in Victoria, Australia 1985-1989. Paediatr Perinat Epidemiol 1994;8:27–40. 10.1111/j.1365-3016.1994.tb00433.x 8153016

[23] Gilbert R, Salanti G, Harden M, et al Infant sleeping position and the sudden infant death syndrome: systematic review of observational studies and historical review of recommendations from 1940 to 2002. Int J Epidemiol 2005;34:874–87. 10.1093/ije/dyi088 15843394

[24] NHS Choices. Sudden infant death syndrome (SIDS). 2017 https://www.nhs.uk/conditions/sudden-infant-death-syndrome-sids/ (accessed Nov 2017).

[25] Garstang J, Ellis C, Griffiths F, et al Unintentional asphyxia, SIDS, and medically explained deaths: a descriptive study of outcomes of child death review (CDR) investigations following sudden unexpected death in infancy. Forensic Sci Med Pathol 2016;12:407–15. 10.1007/s12024-016-9802-0 27503508

[26] Raleigh VS, Hussey D, Seccombe I, et al Ethnic and social inequalities in women’s experience of maternity care in England: results of a national survey. J R Soc Med 2010;103:188–98. 10.1258/jrsm.2010.090460 20436027 PMC2862068

[27] Tomalski P, Moore DG, Ballieux H, et al Separating the effects of ethnicity and socio-economic status on sleep practices of 6- to 7-month-old infants. Learn Individ Differ 2016;46:64–9. 10.1016/j.lindif.2015.12.028

[28] Farooqi S, Perry IJ, Beevers DG Ethnic differences in infant-rearing practices and their possible relationship to the incidence of sudden infant death syndrome (SIDS). Paediatr Perinat Epidemiol 1993;7:245–52. 10.1111/j.1365-3016.1993.tb00402.x 8378167

[29] Gantley M, Davies DP, Murcott A Sudden infant death syndrome: links with infant care practices. BMJ 1993;306:16–20. 10.1136/bmj.306.6869.16 8435569 PMC1676360

[30] Ball HL, Moya E, Fairley L, et al Infant care practices related to sudden infant death syndrome in South Asian and White British families in the UK. Paediatr Perinat Epidemiol 2012;26:3–12. 10.1111/j.1365-3016.2011.01217.x 22150702

[31] Crane D BradICS: Bradford Infant Care Study: a qualitative study of infant care practices and unexpected infant death in an urban multi-cultural UK population. Durham: Durham University, 2014:69–76.

[32] Nelson EA, Taylor BJ, Jenik A, et al International child care practices study: Infant sleeping environment. Early Hum Dev 2001;62:43–55. 10.1016/S0378-3782(01)00116-5 11245994

[33] U.S. Food and Drug Administration. Do not use infant sleep positioners due to the risk of suffocation. https://www.fda.gov/ForConsumers/ConsumerUpdates/ucm227575 (accessed Nov 2017).

[34] Centers for Disease Control and Prevention. Suffocation deaths associated with use of infant sleep positioners — United States, 1997–2011. Morbidity and Mortality Weekly Report (MMWR). 2012 https://www.cdc.gov/mmwr/preview/mmwrhtml/mm6146a1.htm?s_cid=mm6146a1_e 23169313

[35] Griffiths LJ, Tate AR, Dezateux C The contribution of parental and community ethnicity to breastfeeding practices: evidence from the Millennium Cohort Study. Int J Epidemiol 2005;34:1378–86. 10.1093/ije/dyi162 16109734

[36] Griffiths LJ, Tate AR, Dezateux C, et al Do early infant feeding practices vary by maternal ethnic group? Public Health Nutr 2007;10:957–64. 10.1017/S1368980007665513 17381914

[37] Jayaweera H, Quigley MA Health status, health behaviour and healthcare use among migrants in the UK: evidence from mothers in the Millennium Cohort Study. Soc Sci Med 2010;71:1002–10. 10.1016/j.socscimed.2010.05.039 20624665

[38] Wardle H Use of tobacco products. Health Survey for England 2004: the health of minority ethnic groups: The Information Centre, 2006:93–129.

