# Supplementary material for: Ethnic variation in unexplained deaths in infancy, including sudden infant death syndrome (SIDS), England and Wales 2006–2012: national birth cohort study using routine data
Source: J Epidemiol Community Health. 2018 Jul 4;72(10):911–8. doi: 10.1136/jech-2018-210453 (PMC6161655; doi:10.1136/jech-2018-210453)
Supplement: Supplementary file 1 [file jech-2018-210453supp001.pdf]

Supplementary Table S1. Distributions of covariates by ethnic group. Live singleton births at 22+ weeks, England and Wales 2006-2012.

**Ethnic group** / **% of births**	White British	White Non-British	Pakistani	Indian	Bangladeshi	Black African	Mixed Black-African-White	Black Caribbean	Mixed Black-Carib-White	Other / Unspecific	Unstated
**Parent mar/reg**											
Within Marriage	46.2	64.8	95.7	96.2	94.6	61.3	45.7	26.5	20.9	68.3	57.1
Joint, same addr.	36.7	26.8	1.9	2.1	2.7	14.5	26.3	21.7	33.6	16.5	28.5
Joint, diff. addr.	10.9	4.0	1.1	0.9	1.6	12.4	15.8	32.0	28.3	8.5	8.3
Sole registration	6.2	4.4	1.3	0.9	1.2	11.8	12.3	19.8	17.2	6.7	6.1
**Mother COB**											
Non-UK	3.9	80.1	62.9	66.0	78.2	92.7	45.2	36.7	9.4	67.6	26.4
UK	96.1	19.9	37.1	34.0	21.8	7.3	54.8	63.3	90.6	32.4	73.6
**Deprivation, IMD**											
1 = Advantaged	18.0	14.0	3.4	11.4	2.3	3.0	9.3	2.4	8.2	10.6	16.2
2	18.9	16.2	5.4	13.6	4.3	5.4	12.4	4.8	11.1	12.7	18.6
3	19.5	20.0	10.8	20.8	8.8	11.9	17.3	13.0	15.8	17.4	20.1
4	20.2	26.1	25.1	29.9	24.8	29.6	26.1	29.9	25.4	25.9	23.1
5 = Disadvantaged	23.4	23.7	55.3	24.3	59.8	50.1	34.9	49.8	39.4	33.4	22.0
**Gestation, weeks**											
37+	94.5	95.4	94.0	94.0	93.7	93.8	94.5	91.8	93.6	94.5	94.4
32-36	4.7	4.0	5.0	5.1	5.5	4.6	4.5	6.2	5.3	4.6	4.7
22-31	0.8	0.6	1.0	0.9	0.8	1.6	1.0	2.0	1.1	0.9	0.9
**Mother age, years**											
30+	47.1	52.7	37.5	50.5	35.3	54.3	45.9	43.6	34.9	51.1	49.7
25-29	25.8	29.7	37.6	36.6	37.0	29.3	27.7	25.6	25.3	27.8	26.5
20-24	20.0	15.0	22.9	12.2	25.5	13.9	20.4	22.3	27.6	16.9	18.0
<20	7.1	2.6	2.0	0.7	2.2	2.6	6.0	8.5	12.3	4.2	5.8
**Sex**											
Girl	48.7	48.5	49.0	48.7	49.3	49.4	48.8	49.2	48.8	48.6	48.6
Boy	51.3	51.5	51.0	51.3	50.7	50.6	51.2	50.8	51.2	51.4	51.4
**Total**	100	100	100	100	100	100	100	100	100	100	100
**Number of births**	3,009,144	340,515	180,265	132,646	62,944	154,071	29,313	47,503	46,445	344,186	287,732

Parent mar/reg, parents' marital/registration status; COB, country of birth; Gestation, gestational age at birth.

Data for Figure 1 in 'Ethnic variation in unexplained deaths in infancy, including sudden infant death syndrome (SIDS), England and Wales 2006-2012: national birth cohort study using routine data'.

Supplementary Table S2. Unexplained death in infancy by recorded cause. Deaths by age at death (completed months). Deaths per 1000 live singleton births by year of birth. Live singleton births at 22+ weeks, England and Wales 2006-2012.

**Recorded cause**		**Sudden Infant Death Syndrome**		**Unascertained**	
**Age at death (months)**	**N deaths**	**N deaths**	**%**	**N deaths**	**%**
0	274	162	59.1%	112	40.9%
1	430	290	67.4%	140	32.6%
2	302	222	73.5%	80	26.5%
3	175	129	73.7%	46	26.3%
4	126	88	69.8%	38	30.2%
5	83	60	72.3%	23	27.7%
6	51	33	64.7%	18	35.3%
7	37	26	70.3%	11	29.7%
8	36	24	66.7%	12	33.3%
9	19	11	57.9%	8	42.1%
10 or 11	26	17	65.4%	9	34.6%
**Total**	1,559	1,062	68.1%	497	31.9%
**Year of birth**	**N births**	**N deaths**	**Rate/1000**	**N deaths**	**Rate/1000**
2006	631,676	159	0.2517	72	0.1140
2007	646,862	179	0.2767	70	0.1082
2008	663,899	158	0.2380	78	0.1175
2009	659,783	152	0.2304	78	0.1182
2010	671,249	129	0.1922	77	0.1147
2011	675,057	141	0.2089	68	0.1007
2012	686,238	144	0.2098	54	0.0787
**Total**	4,634,764	1,062	0.2291	497	0.1072

Data for Figure 2 in ‘Ethnic variation in unexplained deaths in infancy, including sudden infant death syndrome (SIDS), England and Wales 2006-2012: national birth cohort study using routine data’.
